# Enrichment of Pasteurized Dairy Product and Brownie With Edible Insect ( *Tenebrio molitor* ) to Analyze Acceptance Using Check‐All‐That‐Apply Methodology

**DOI:** 10.1002/fsn3.70925

**Published:** 2025-10-30

**Authors:** Marta Ros‐Baró, Marta Capellas Puig, Gemma Chiva‐Blanch, Diana A. Díaz‐Rizzolo, Alicia Aguilar Martínez, Montserrat Jorba Rafart, Irina Chiriac, Mar Blanco Rogel, Montserrat Pujolà Cunill, Patricia Casas‐Agustench, Anna Bach‐Faig

**Affiliations:** ^1^ NUTRALiSS Research Group, Faculty of Health Sciences Open University of Catalonia (UOC) Barcelona Spain; ^2^ Centre D'innovació, Recerca i Transferència en Tecnologia Dels Aliments (CIRTTA), TECNIO‐CERTA‐UAB, Departament de Ciència Animal i Dels Aliments, Facultat de Veterinària Universitat Autònoma de Barcelona Bellaterra Spain; ^3^ Centro de Investigación Biomédica en Red de la Fisiopatología de la Obesidad y Nutrición (CIBEROBN), Instituto de Salud Carlos III (ISCIII) Madrid Spain; ^4^ Division of Endocrinology, Nutrition Obesity Research Center, Diabetes Research Center Columbia University Irving Medical Center New York New York USA; ^5^ Faculty of Health Sciences Open University of Catalonia (UOC) Barcelona Spain; ^6^ LEITAT Technological Center Terrassa Spain; ^7^ Department of Agri‐Food Engineering and Biotechnology Universitat Politècnica de Catalunya BarcelonaTech Barcelona Spain; ^8^ School of Health Professions, Faculty of Health University of Plymouth Plymouth UK

**Keywords:** alternative proteins, consumer acceptance, edible insects, novel foods, sensory profile, *Tenebrio molitor*

## Abstract

Insect proteins, especially from *Tenebrio molitor* (*TM*), are emerging as sustainable alternatives to conventional proteins. This study aimed to design and evaluate *TM*‐enriched food formulations for individuals with increased protein needs, assessing their nutritional, physicochemical, and sensory properties, and exploring feasibility. Two sensory evaluations were conducted using the Check‐All‐That‐Apply (CATA) method. The first (CATA1) assessed the direct incorporation of *TM* powder (32 g/100 g) into a pasteurized dairy product (D) with hazelnut (DTM_1_H), hazelnut–vanilla (DTM_1_HV), or vanilla (DTM_1_V) aroma. DTM_1_HV reached 52.4% acceptability. The second (CATA2) evaluated brownies with three formulations: D and *TM* powder (BDTM_1_), *TM* powder (BTM_1_), and *TM* powder and *TM* protein hydrolysate (BTM_2_). Hydrolysate‐based formulation had a significantly higher protein‐to‐lipid ratio, offering a better nutritional profile than those with only *TM* powder. Physicochemical analysis showed BTM_2_ improved cohesiveness and elasticity (*p* < 0.05), while higher *TM* powder (BTM_1_) increased hardness and bitterness (*p* < 0.05). In BTM_2_, bitterness suppressed sweetness and reduced liking (*p* < 0.05). Correlation analysis showed strong positive associations between crumb and crust color acceptability (*r* = 0.72–0.80), and between sponginess in hand and in mouth (*r* = 0.65–0.91). Sweetness also correlated positively with global aspect (*r* = 0.53–0.67). These findings support that hazelnut–vanilla aroma enhances dairy product acceptability, while *TM* protein hydrolysate contributes to improved nutritional and sensory profiles in high‐protein formulations.

## Graphical abstract

BDTM_1_, Brownie with pasteurized dairy product and *Tenebrio molitor* powder; BTM_1_, Brownie with *Tenebrio molitor* powder; BTM_2_, Brownie with *Tenebrio molitor* powder and *Tenebrio molitor* protein hydrolysate; DTM_1_H, Pasteurized dairy product with *Tenebrio molitor* powder and hazelnut aroma; DTM_1_HV, Pasteurized dairy product with *Tenebrio molitor* powder and hazelnut and vanilla aroma; DTM_1_V, Pasteurized dairy product with *Tenebrio molitor* powder and vanilla aroma; *Tenebrio molitor* (T); Pasteurized dairy product (D).

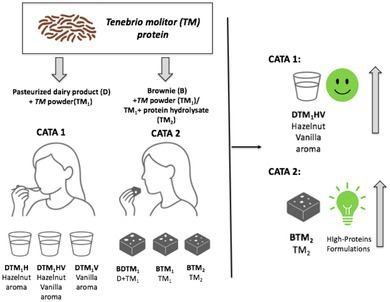

## 1 Introduction

Current food systems face increasing pressure to evolve toward models that better support both human health and planetary sustainability goals (Panel 2020). Evidence suggests that global food production is already exceeding key environmental thresholds—especially in water use and ocean health—posing significant challenges in regions such as Spain (Vargas‐Amelin and Pindado 2014). To address future food security challenges, a shift in food production, consumption patterns, and waste reduction is essential. This shift must promote environmental sustainability, affordability, and health‐conscious solutions for a projected global population of 9.7 billion by 2050 (Gu et al. 2021).

One significant challenge is managing the anticipated 70% to 80% increase in protein demand, specifically for animal protein, between 2030 and 2050 (Willett et al. 2019). In response, alternative protein sources—including cultured meat, plants, insects, algae, fungi, and unicellular organisms—are being explored for their sustainability potential (Salter and Lopez‐Viso 2021). Insects, which have been part of human diets for millennia (especially in Asian and Central and South American cultures), have gained scientific interest as a food source since 2013, following recommendations from the United Nations Food and Agriculture Organization (FAO) to explore their low environmental footprint (van Huis et al. 2013).

Compared to traditional livestock, edible insects have a 40%–60% lower impact on land, water, and greenhouse gas emissions (Govorushko 2019; Ros‐Baró, Casas‐Agustench, et al. 2022; Rumpold and Schlüter 2013; van Huis et al. 2013). Regulatory bodies, such as the European Union, recognize edible insects as novel foods (UE 2015/2283) (Nuţă 2017), and the European Food Safety Authority (EFSA) has issued positive opinions on five species for human consumption: *Tenebrio molitor* (*TM*) (EFSA Panel on Nutrition et al. 2021a), *Locusta migratoria* (EFSA Panel on Nutrition et al. 2021b) *Acheta domesticus* (EFSA Panel on Nutrition et al. 2022b), and *Alphitobius diaperinus* (EFSA Panel on Nutrition et al. 2022a). Among these, *TM* stands out for its nutritional richness. Its larvae contain 49.1% protein, along with essential fatty acids and minerals, making them a promising protein source (Payne et al. 2016). Insect proteins such as *TM* exhibit amino acid profiles comparable to milk and meat (Perez‐Santaescolastica et al. 2023) supporting postprandial muscle protein synthesis similar to high‐quality animal proteins (Hermans et al. 2021). *TM* also offers additional nutritional benefits by improving the n6/n3 fatty acid ratio and increasing dietary fiber content, both factors associated with beneficial effects on cardiovascular health (Badimon et al. 2019; Gonzalez‐Becerra et al. 2023).

*TM*'s amino acid composition holds particular promise for muscle function and maintenance. Leucine, a branched‐chain essential amino acid, plays a central role in the regulation of muscle protein synthesis (MPS), particularly under conditions associated with increased risk of muscle mass loss such as aging, caloric restriction, or high physical demands. Its ability to activate the mechanistic target of rapamycin (mTOR) signaling pathway makes leucine a key anabolic trigger in skeletal muscle (Atherton et al. 2017). Reaching a per‐meal leucine threshold of approximately 2–3 g in young adults, and 3–4 g in older individuals, is necessary to maximally stimulate MPS and overcome anabolic resistance (Breen and Phillips 2011; Churchward‐Venne et al. 2014). This can typically be achieved through the intake of 25–30 g of high‐quality protein such as whey, which contains 8–11 g of leucine per 100 g (Tang et al. 2009). Studies report that *TM* protein contains approximately 6.5–8.0 g of leucine per 100 g of protein, which is comparable to some conventional animal proteins (Meyer‐Rochow et al. 2021; Oliveira et al. 2024). This leucine density suggests that *TM* could effectively contribute to reaching the leucine threshold for MPS stimulation, particularly when used in isolated or concentrated protein forms. Consequently, insect‐based proteins such as *TM* may offer a viable alternative for maintaining muscle health in vulnerable populations and in sports nutrition, while aligning with environmental sustainability goals.

Despite *TM*'s potential to support muscle health and sustainability goals, its integration into mainstream diets faces significant obstacles. While interest in insect‐based nutrition is growing, particularly among consumers concerned with health and the environment, acceptance in Western markets remains limited (Halloran et al. 2015; Jagtap et al. 2021; Orkusz 2021). Familiarity with insect‐based products, particularly in less recognizable forms (e.g., powders or protein bars) (Conway et al. 2024; Okaiyeto et al. 2024) may enhance acceptance. Therefore, processing methods also play a crucial role in mitigating negative perceptions (Borges et al. 2022; Draszanowska et al. 2024). Research in Mediterranean regions indicates that disgust and food safety concerns are primary deterrents. Introducing insect protein into popular foods, such as brownies, cookies, and bread, could further aid acceptance by creating appealing, familiar formats (Ros‐Baró, Sánchez‐Socarrás, et al. 2022). To support broader acceptance, it is essential to conduct extensive studies evaluating changes in sensory attributes and their impact on product appeal, alongside physicochemical characteristics. That is particularly important given the fact that higher *TM* concentrations often lead to reduced consumer acceptability (Amoah et al. 2023; Zielińska et al. 2021). In this context, understanding how formulation strategies can enhance both nutritional value and palatability becomes crucial (Rocha et al. 2025). These findings underscore the importance of aligning sensory appeal with consumer expectations, particularly in light of the growing interest in protein‐enriched products derived from insect sources. In support of this trend, recent research has increasingly explored the incorporation of insect powder into baked goods across various regions. For example, Lucas et al. (2004) suggested that demand for protein‐enriched dairy products, such as those made with *TM* and containing bioactive peptides, is expected to grow to meet daily nutritional requirements (Azzollini 2017; Kröncke and Benning 2023). Further research in Spain has primarily focused on the use of insect powder in baked goods (Amoah et al. 2023), such as cupcakes, chocolate chip cookies (Aguilera et al. 2021) cookies (Ortolá et al. 2022) and bread (González et al. 2019). Notably, only two studies of Amoah et al. (2023) review have explored formulations with over 20% edible insect content exceeding 20% (Amoah et al. 2023).

Among the studies reviewed in this research, two studies (Hernandez et al. 2019; Karwacka et al. 2024) incorporated up to 5 g of edible insect powder ( *Acheta domesticus* and *TM*) into the formulation of a yogurt‐based product and analyzed the properties and consumer acceptability. Additionally, three studies (Gurdian et al. 2021a, 2021b; Ho et al. 2022) investigated the inclusion of *TM* in brownies. One of these studies (Ho et al. 2022) highlighted the need to increase the percentage of insect protein to more accurately evaluate its acceptability. In the remaining studies (Gurdian et al. 2021a, 2021b), a check‐all‐that‐apply (CATA) methodology was utilized to assess consumer acceptance patterns, supplemented with contextual information on the nutritional and environmental benefits of brownies formulated with *Acheta domesticus* . These formulations may support the inclusion of higher amounts of *TM* while maintaining acceptable product quality. Across these studies (Amoah et al. 2023), the highest reported concentration of *TM* powder was 15%. Notably, none of the articles reviewed thus far have incorporated hydrolysates into the formulation, which opens up a new avenue for exploring this type of ingredient in the development of recipes such as brownies. *TM* protein hydrolysate remains highly relevant for future food and nutrition research. Protein hydrolysates, even at moderate hydrolysis levels, can provide improved digestibility, increased solubility, and enhanced functional properties such as emulsification and foaming capacity, which are valuable in complex food matrices (Mshayisa et al. 2022). In addition, the use of *TM* hydrolysates aligns with sustainability goals by valorizing insect protein into high‐value functional ingredients suitable for inclusion in foods targeted to vulnerable populations such as the elderly, athletes, or individuals with malabsorption issues (Yoon et al. 2019). Their potential to be tailored through controlled enzymatic treatment also opens the door to fine‐tuning their bioavailability and peptide profile for specific health outcomes (de Castro et al. 2018).

Building on gaps identified in prior formulations, particularly the limited use of *TM* hydrolysates and the challenges associated with increasing insect protein concentrations while preserving product quality, this study aimed to explore the potential of *TM* in real food matrices. To address these challenges, two sensory evaluations were conducted using the Check‐All‐That‐Apply (CATA) methodology. The first (CATA1) assessed the direct incorporation of *TM* powder into a pasteurized dairy product, while the second (CATA2) evaluated the feasibility and acceptability of *TM* protein in a brownie formulation. The specific objectives were: (1) to design *TM*‐enriched food formulations targeting individuals with elevated protein needs; (2) to evaluate their nutritional, physicochemical, and sensory properties within a sustainable protein framework; and (3) to compare the sensorial properties of *TM*‐enriched food formulations using *TM* hydrolysate or powder in brownies, focusing on its influence on texture, acceptability, and physicochemical performance. This approach introduces a novel contribution by incorporating *TM* hydrolysate into a bakery product—a strategy not previously explored in the literature. The findings may inform the development of sustainable foods tailored to populations with specific nutritional requirements.

## 2 Material and Methods

### 2.1 Ingredients

The ingredients used in the natural pasteurized dairy product (D) with edible insect, and brownie with total substitution of original‐recipe wheat flour with *TM*‐based formulations were: *TM* powder (TM_1_) and TM_1_ with a *TM* protein hydrolysate (TM_2_) INSEKT LABEL BIOTECH S.L., Basque Country, Spain, D product (CALIDAD PASCUAL S.A.U, Madrid, Spain), sweetener Dulzol (CASA SANTIVERI S.L, Barcelona, Spain), hazelnut aroma (ESSENTIALS S.L, Barcelona, Spain), and vanilla aroma (DR. OETKER IBÉRICA S.L, Bielefeld, Germany). TM_1_ had a particle size of ≤ 2 mm, medium brown color, smooth texture, and a cereal‐like smell. Samples of *TM* powder were distributed, packaged in 2 kg plastic bags each, and stored at ambient temperature until use. INSEKT LABEL BIOTECH S.L. guaranteed compliance with current regulations on good practice and food hygiene, and conducted all legally required tests at the microbiological and toxic metals safety level throughout the production process. INSEKT LABEL BIOTECH S.L. developed TM_1_ and the experimental *TM* protein hydrolysate. *TM* protein hydrolysate is a concentrated enzymatic hydrolysate with a molasses‐type appearance. TM_1_ may contain gluten and soy, and may cause allergic reactions in consumers with known allergies to shellfish and shellfish products or to dust mites. All remaining ingredients were purchased from local grocery stores in the Barcelona area.

### 2.2 Preparation of Food Products With *TM* ‐Based Ingredients

The ingredients for each formulation and their concentrations are listed in Table 1. The amount of protein source in each formulation was adjusted to obtain the same final quantity of leucine in the food product. Before use, TM_1_ particle size was homogenized to 1 mm using a sieve.

**TABLE 1:** Ingredients (g) used in different formulations in CATA 1 and CATA 2 per 100 g of edible product.

Ingredient	D with *TM* powder (TM_1_)			Brownie with total substitution of wheat flour with *TM* ingredients		
	DTM_1_H	DTM_1_V	DTM_1_HV	BDTM_1_	BTM_1_	BTM_2_
Pasteurized dairy product	66.2	66.5	66.4	24.8	0.0	0.0
*TM* powder	32.0	31.9	31.9	33.1	40.2	26.5
*TM* protein hydrolysate				0.0	0.0	18.5
Sweetener	1.5	1.5	1.5			
Hazelnut aroma	0.3	0.0	0.1			
Vanilla aroma		0.1	0.1			
Egg				19.9	21.1	19.4
Egg yolk					3.6	3.3
Egg white				16.5	10.7	9.9
Sugar				0.0	6.7	6.1
Cocoa powder				4.2	4.5	4.1
Butter					13.2	12.2
Baking powder				1.3		
Salt				0.2		

Abbreviations: BDTM_1_, Brownie with pasteurized dairy product and *Tenebrio molitor* powder; BTM_1_, Brownie with *Tenebrio molitor* powder; BTM_2_, Brownie with *Tenebrio molitor* powder and *Tenebrio molitor* protein hydrolysate; DTM_1_H, Pasteurized dairy product with *Tenebrio molitor* powder and hazelnut aroma; DTM_1_HV, Pasteurized dairy product with *Tenebrio molitor* powder and hazelnut and vanilla aroma; DTM_1_V, Pasteurized dairy product with *Tenebrio molitor* powder and vanilla aroma.

#### 2.2.1 D With *TM* Powder (CATA1)

**FIGURE 1:**
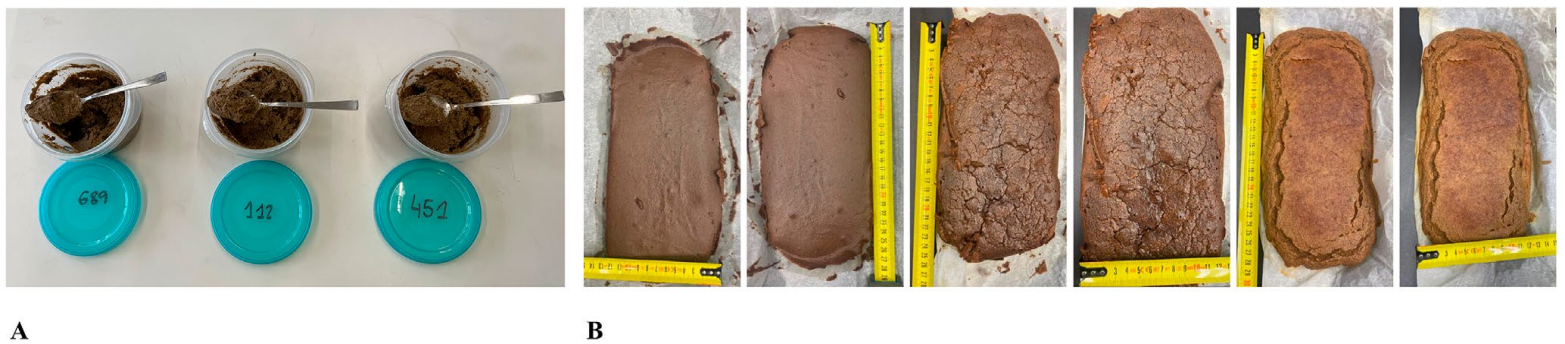
The images are presented in the following order (A) Top view of D with *TM* powder: DTM_1_H, Pasteurized dairy product with *Tenebrio molitor* powder and hazelnut aroma; DTM_1_HV, Pasteurized dairy product with *Tenebrio molitor* powder and hazelnut and vanilla aroma; DTM_1_V, Pasteurized dairy product with *Tenebrio molitor* powder and vanilla aroma. (B) BTM_1_, Brownie with *Tenebrio molitor* powder; BTM_2_, Brownie with *Tenebrio molitor* powder and *Tenebrio molitor* protein hydrolysate and BDTM_1_, Brownie with pasteurized dairy product and *Tenebrio molitor* powder.

Three formulations differing in the type of flavor were prepared: D with *TM* powder and hazelnut flavor (DTM_1_H), D with *TM* powder and hazelnut and vanilla aroma (DTM_1_HV), D with *TM* powder and vanilla flavor (DTM_1_V). Ingredients were mixed in food containers and stored at 18°C until sensory analysis or at 4°C until physicochemical analysis (Figure 1).

#### 2.2.2 Brownie With Total Substitution of Wheat Flour With *TM* ‐Based Ingredients (CATA2)

Three different formulations of brownie were prepared according to Dunnewind et al. (2003) with slight modifications and total substitution of wheat flour with *TM*‐based ingredients (Table 1 and Figure 1). Total substitution of wheat flour with *TM* powder was carried out in order to evaluate its full impact on the physicochemical, nutritional, and sensory properties of the final product, as opposed to the partial substitutions commonly reported in previous studies (Gantner et al. 2022). In the same line, a formulation was sought that would ensure optimal acceptability of the hydrolysate ingredient.

The brownie with total substitution of wheat flour with *TM* powder and D (BDTM_1_) imitated a yogurt cake. The recipe combined eggs, D, and dry ingredients such as insect powder and orange zest, which produced a creamier and fluffier texture after baking at 170°C for 40 min.

The brownie prepared with total substitution of wheat flour with *TM* powder (BTM_1_) followed a traditional moist brownie recipe with almonds and chocolate. The preparation consisted of mixing butter, eggs, egg yolks, and cocoa, gradually incorporating insect powder and then a meringue made with egg whites and sugar. This mixture was baked at 170°C for 25 min.

The brownie with 26.5% *TM* powder and 18.5% *TM* protein hydrolysate (BTM_2_) replaced wheat flour with *TM* powders and *TM* protein hydrolysate, using the same method as BTM_1_.

According to the Federal Agency for the Safety of the Food Chain (Scientific Committee of the FASFC 2014), the daily intake of 45 g of freeze‐dried insects containing an average of 6% chitin poses no health risk. In our product, the portion size consumed by panelists was approximately 20–30 g, which contained about 32.6% *TM* powder. Since *TM* powder had a known chitin content of 5%–7% (Noyens et al. 2024), the resulting chitin intake from our product remained significantly below the established safe limit, presenting no health concerns.

### 2.3 Nutritional Value

Nutritional assessment of the product was calculated based on the Easydiet nutritional assessment program (Malinowska 2022) endorsed by the University of Barcelona (https://www.easydiet.es/). In this study, leucine was the only amino acid analyzed due to its critical role in future formulations targeting populations with nutritional deficits, such as older adults and individuals engaged in regular physical activity (Katsanos et al. 2006).

### 2.4 Physicochemical Properties

The moisture content was analyzed in duplicate using the AOAC 925.10 oven drying method. The pH was determined using a Micro‐pH 2001 pHmeter (CRISON, Barcelona, Spain). Water activity was analyzed using an Aqualab 3TE dew point hygrometer (DECAGON DEVICES, Pullman, USA). Color of the crumb was measured with a CR‐400 Chroma Meter colorimeter (KONICA MINOLTA INC., Osaka, Japan) using a D65 light source and a 10° angle observer. The results were expressed in the CIE color space L* a* b*, where L* is the lightness, with values from 0 (black) to 100 (white), a* is the chromatic component from green (negative values) to red (positive values), and b* from blue (negative values) to yellow (positive values). Analyses were performed in duplicate except for color, which was measured in triplicate.

The initial (raw) and final (cooked) weights of the brownies were recorded, and baking loss (%) values were calculated as the percentage difference between batter weight and brownie weight according to an equation by Heo et al. (2019). The baking loss procedure was performed in triplicate.

#### 2.4.1 Texture Analysis

D with insect powder (*TM*): A back‐extrusion test was performed in triplicate using a texture analyzer (TA.XT2 model, STABLE MICRO SYSTEMS, Surrey, UK) equipped with a flat cylindrical probe with a diameter of 35 mm. By applying a constant velocity of 1 mm/s and a depth of 30 mm, and withdrawing the probe at the same speed to the starting position, the force‐time curves were obtained.

In the brownies, texture profile analysis was performed in triplicate on samples measuring 2 × 2 cm with a 10 cm flat probe using a TA TX2 texture analyzer. Probe speed was set to 1 mm/s to compress the center of the brownie sample to 40% of its original height. Time between compressions was 5 s. All the parameters analyzed in the study are presented in Table 2.

**TABLE 2:** Attributes evaluated by consumers using CATA questions for sensory characterization of D with insect powder (*TM*).

Sensory characteristics	Descriptor	D with insect powder (TM_1_)	Brownie with total substitution with insect powder (TM_1_; TM_2_)
Appearance	Phase separation (syneresis)	X	
	Viscosity	X	
	Color	X	
	Crumb color		X
	Crust color		X
Flavor (Taste and Smell)	Sweetness	X	X
	Bitterness	X	X
	Acidity	X	
	Astringency	X	
	Aroma		X
Texture	Hard	X	X
	Floury	X	
	Creamy	X	
	Spongy in mouth		X

*Note:* The data table of CATA analysis contains the sensory attributes in the rows, the assessors and products in the columns. This table signifies X an attribute has been identified by an assessor, while not X indicates that the assessor did not perceive the attribute in the sample.

Abbreviations: D, pasteurized dairy product; TM_1_, *Tenebrio molitor* powder; TM_2_, powder and *Tenebrio molitor* protein hydrolysate.

### 2.5 Sensory Evaluation

#### 2.5.1 Study Participants

All evaluations were conducted following the CATA methodology. CATA questionnaires involve presenting participants with a list of phrases or words, from which they select all that they deem appropriate. Due to their versatility, simplicity, speed, and effectiveness, CATA experiments have gained popularity in recent years. This method is now widely used for sensory analysis of products by both trained panels and consumers. Their quick and straightforward nature requires less cognitive effort from assessors, making consumer‐driven sensory characterization valuable for product optimization. Assessors in CATA experiments identify sensory attributes present in the sample from a provided list. This list typically comprises sensory characteristics of the product, along with hedonic terms, emotions, and non‐sensory properties. Assessors can freely select from the list without constraints on the number of terms chosen (Ares et al. 2010). The terms used in both CATA questionnaires are listed in Table 2.

The consumer test CATA1 was conducted with 21 volunteer participants (8 men and 13 women), all over the age of 60. The second consumer test, CATA2, was carried out with 25 participants (8 men and 17 women) ranging in age from 19 to 73.

The participants for CATA1 were recruited via a telephone questionnaire and invited to participate as sensory test panelists for food products containing edible insect powder. Participants for CATA2 were recruited during the 4th Science and Cooking World Congress held in Barcelona on November 13, 2024 (https://scienceandcookingworldcongress.com/en/).

**FIGURE 2:**
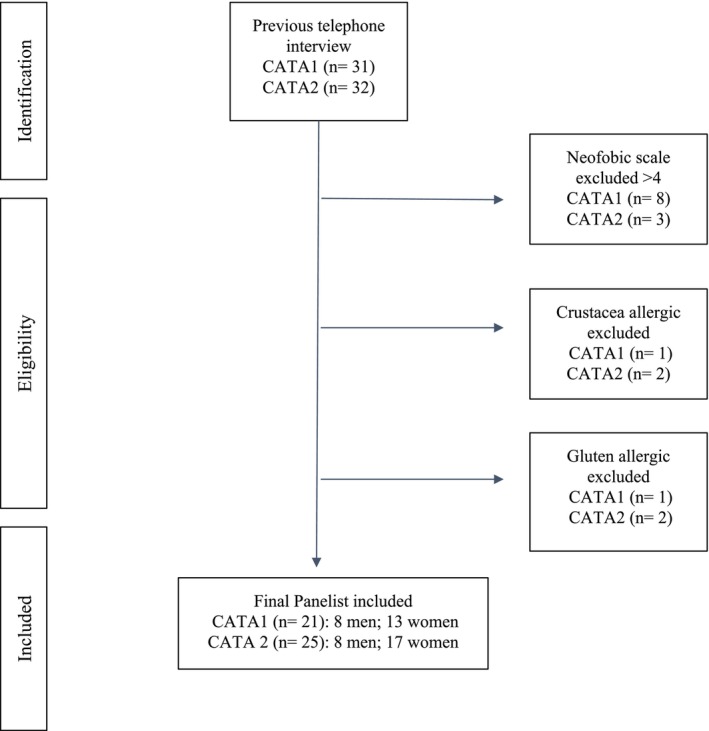
Flow chart of eligibility criteria of CATA1 and CATA2, respectively.

The participants included in CATA1 and CATA2 had no food allergies, no notable illnesses, and the neophobia test gave a value of < 4. Figure 2 presents the flow chart of eligibility criteria of both CATA tests. The study adhered to the Declaration of Helsinki for Medical Research involving Human Subjects and received approval from the Ethics Committee (Exp.: ce23‐TE33) of the Universitat Oberta de Catalunya (UOC). Prior to conducting CATA1 and CATA2, participants were informed about the general aim of the study and the evaluation procedure. A final phone call specified the time and place of the CATA1 and CATA2 tests and informed the participants that consent forms and image rights would be signed on the day of the CATA1 and CATA2 tests. Panelists were asked not to eat/drink/smoke or wear perfume for at least 3 h before the evaluation session. Both assessments were conducted by an inexperienced panel to evaluate the product's acceptability and organoleptic characteristics in each instance.

#### 2.5.2 Neophobia Test

To assess food neophobia, a survey with 10 items, including reverse‐coded items, was conducted. Items 1, 4, 6, 9, and 10 were evaluated as follows: 1 = Completely agree, 2 = Moderately agree, 3 = Slightly agree, 4 = Neither agree nor disagree, 5 = Slightly disagree, 6 = Moderately disagree, and 7 = Strongly disagree. Items 2, 3, 5, 7, and 8 were evaluated in the opposite way, where 1 = Completely disagree and 7 = Completely agree. The identification of neophobia was defined as > 1 standard deviation from the mean, as reported in the bibliography (Pliner and Hobden 1992).

#### 2.5.3 Acceptance and Preference Test

Acceptability of the formulations was evaluated using a 9‐point hedonic scale, where a score of 1 represented dislike extremely and a score of 9 meant like extremely (Jones et al. 1955). At the end of the test, panelists were asked to rank the samples in order of preference. The study adhered to a completely randomized design. Tests were carried out with appropriate temperature and lighting to ensure the comfort and privacy of the panelists (Stone et al. 2020). Formulations were presented at 23°C in odorless plastic containers coded with random three‐digit codes. For sustainability and safety reasons, the product packaging, glasses, and spoons were made from recycled materials. The labels on the provided boxes were covered to ensure that the participating researchers remained blinded to the supplementation assignments of the study participants.

### 2.6 Statistical Analysis

Statistical analyses included the calculation of Cronbach's alpha to assess the internal consistency of a preliminary neophobia analysis. One‐way analysis of variance (ANOVA), a statistical method used to compare the means of three or more groups to determine if at least one significantly differs from the others, was applied followed by Tukey's post hoc test for multiple pairwise comparisons at *p* ≤ 0.05 to detect significant differences among attributes and product formulations. Spearman's correlation coefficient was employed to examine relationships between variables, while chi‐square tests assessed potential differences in the evaluation of overall product appearance. Instrumental physicochemical properties were also analyzed via ANOVA using two or three replicate measurements per property, with no observed within‐product variability; Tukey's HSD test was used to compare BTM_1_–BTM_2_ and BDTM_1_–BTM_1_ when significant differences were identified (*p* ≤ 0.05). Principal component analysis (PCA) was conducted to reduce dimensionality and identify underlying patterns by transforming correlated variables into uncorrelated components, capturing the greatest variance to improve interpretability and visualization. Data processing included the calculation of means, normalization, covariance matrix computation, and PCA transformation, with the biplot effectively summarizing the data structure and relationships among variables. All analyses were conducted using R software (version 4.3.2; R Foundation for Statistical Computing, Vienna, Austria).

## 3 Results and Discussion

### 3.1 Nutritional Value

Calculations were based on the nutritional values labeled on the packaging of the ingredients used (Table 3). *TM* powder exhibited a higher (content of protein 41.2% and fat 30.7% respectively) compared with wheat flour (Matiza Ruzengwe et al. 2022). This suggests that edible insect powder could be considered an enriching product in terms of these macronutrients for food formulations (Pal and Roy 2014). Insect meal demonstrated high protein quality and digestibility, with leucine levels ranging 3.6–4.2 g/100 g—values consistent with or exceeding those reported by Zielińska et al. (2015). This supports its potential as a nutrient‐dense ingredient, particularly when compared with conventional protein sources or formulations assessed in similar studies.

**TABLE 3:** Macronutrients, fiber, and salt composition of the ingredients used and manufactured products (g/100 g).

Nutrients	D	*TM* (powder)	*TM* (hydrolyzed)	D with insect powder (TM_1_)^b^			Brownie with total substitution with edible insect (TM_1_; TM_2_)^b^		
				DTM_1_H	DTM_1_V	DTM_1_HV	BDTM_1_	BTM_1_	BTM_2_
Lipids (g)	1.9^a^	33^a^	7.6^a^	12.4	12.4	12.4	14.3	27.8	23.5
Saturated (g)	1.4^a^	8^a^	NA	3.6	3.6	3.6	3.8	10.9	9.2
Polyunsaturated (g)	NA	7.1^a^	NA	2.4	2.4	2.4	2.8	3.7	2.6
Carbohydrates (g)	6.9^a^	4.2^c^	2.4^c^	6	6	6	3.4	12.1	10.8
Protein (g)	3.4^a^	52.2^a^	38.8^a^	19.9	19.9	19.9	23.6	25.1	24.6
Leucine (g)	0.34^a^	4.3^a^	2.99^a^	1.5	1.5	1.5	2	2.1	2
Fiber (g)	NA^a^	3.7^a^	1^a^	1.2	1.2	1.2	1.3	1.5	1
Salt (g)	0.12^a^	0.3^a^	NA	0.3	0.3	0.3	0.5	0.2	0.1
Protein/lipid	1.8	1.6	5.1	1.6	1.6	1.6	1.7	0.9	1
%Water^d^	87.48 ± 0.04	5.86 ± 0.08	50.19 ± 0.01	61.84 ± 0.05	57.13 ± 3.85	61.84 ± 1.6	53.19 ± 1.68	35.25 ± 0.54	34.59 ± 1.54

Abbreviations: BDTM_1_, Brownie with pasteurized dairy product and *Tenebrio molitor* powder; BTM_1_, Brownie with *Tenebrio molitor* powder; BTM_2_, Brownie with *Tenebrio molitor* powder and *Tenebrio molitor* protein hydrolysate; D, Pasteurized dairy product; DTM_1_H, Pasteurized dairy product with *Tenebrio molitor* powder and hazelnut aroma; DTM_1_HV, Pasteurized dairy product with *Tenebrio molitor* powder and hazelnut and vanilla aroma; DTM_1_V, Pasteurized dairy product with *Tenebrio molitor* powder and vanilla aroma; *TM* hydrolysate, *Tenebrio molitor* protein hydrolysate; *TM* Powder, *Tenebrio molitor* powder.

^a^ Values from the product label.

^b^ Values calculated from the ingredients.

^c^ Values calculated by difference.

^d^ Analyzed values (Mean ± SD).

The formulated products could be labeled as high in protein, as per Regulation (EC) No 1924/2006 of the European Parliament and the Council of 20 December 2006 on nutrition and health claims made on foods. According to this regulation, a food product can be considered high in protein if at least 20% of its energy content is derived from proteins, which is the case with the tested formulations (Bettencourt‐Câmara et al. 2016).

As sustainable protein supplements rise in popularity, insects such as *TM* could represent a pivotal step forward in addressing both nutritional and environmental challenges. Their integration into dietary practices may provide a novel pathway toward meeting global protein needs sustainably. Among animal‐based proteins, cooked meats such as chicken breast, lean beef, and salmon contain leucine levels of 1.69 ± 0.04 g/100 g. Dairy products and eggs provide a range of leucine levels, with parmesan cheese at 1.51 ± 0.06 g/100 g. Plant‐based sources, including cooked lentils, soybeans, and quinoa, offer leucine contents of 0.88 ± 0.02 g/100 g. Nuts, represented by almonds, contain 1.48 ± 0.04 g/100 g. Lastly, protein supplements such as whey protein powder exhibit the highest leucine content, averaging 8.70 ± 0.20 g/100 g, making them an efficient concentrated source of this amino acid (Norton et al. 2012). The present study further explores the potential of insect‐based proteins by evaluating both *TM* powder and *TM* protein hydrolysate in brownie formulations. Given the increasing interest in sustainable protein sources with favorable amino acid profiles—particularly leucine—*TM* emerges as a viable alternative. Although not as concentrated as whey protein, *TM*‐based ingredients offer leucine levels comparable to many whole‐food sources, such as nuts and dairy products. Furthermore, the study highlights how processing (i.e., hydrolysis) impacts the compositional and sensory characteristics of the final product, suggesting that both *TM* powder and hydrolysate can contribute not only to protein enrichment but also to consumer‐acceptable functional food development. These findings support the integration of *TM*‐derived ingredients into food systems as a nutritionally relevant and sustainable strategy for addressing global protein needs (aimed at target populations such as older adults, athletes, or people with protein deficiency).

The search for new, more sustainable sources of long‐chain amino acids continues to gain attention due to the increasing demand for environmentally friendly protein alternatives. Studies have demonstrated that supplementing the diet with 2 g of leucine, combined with weekly physical exercise, significantly enhances muscle synthesis (Murphy et al. 2016). In this context, the present study aimed to develop formulations containing approximately 2 g of leucine, which could potentially address this nutritional demand. Future research should further investigate the amino acid profiles—particularly leucine content—and bioavailability of *TM* powder and hydrolysates, as well as their efficacy in supporting muscle protein synthesis in functional food applications (van Huis 2020b). Incorporating insect‐derived protein in a familiar, palatable matrix like a brownie enables a functional delivery of essential amino acids—particularly leucine—while also promoting dietary diversity and supporting sustainable protein sources. This approach combines nutritional functionality with environmental sustainability, aligning with global health and food system goals.

The leucine content in *TM*‐based ingredients closely aligns with the dietary intake recommendations established by the FAO (Leser 2013). These findings support the potential of *TM* powder and hydrolysate as viable sources of essential amino acids, specifically leucine, echoing the conclusions drawn in the FAO/WHO/UNU Consultation (2011). Furthermore, the findings from this study are consistent with those reported by Mshayisa et al. (2022) (Mshayisa et al. 2022) who demonstrated that the incorporation of *TM* powder and *TM* protein hydrolysate treatments affects techno‐functional properties and broadens the potential application range of insects‐based ingredients. In terms of lipid content, we observed a range between studies: the lowest revised value reported was 14.29% (Mazurek et al. 2024) while the highest value was 34.54% (Ghosh et al. 2017). The lipid content of the powder we utilized was measured at an intermediate value of 33%, positioning it well within this range. The lipid profile of *TM* is of nutritional interest due to its richness in polyunsaturated fatty acids (PUFAs), particularly linoleic acid (C18:2, ω‐6) and, in some strains, detectable levels of α‐linolenic acid (C18:3, ω‐3). These essential fatty acids play critical roles in human health, contributing to cardiovascular protection, cognitive development, and anti‐inflammatory responses (Orkusz 2021; Arnold Van Huis 2020a). The dietary ratio of ω‐6 to ω‐3 fatty acids is a key determinant of metabolic balance, and the incorporation of *TM* into food systems may offer novel ways to enhance PUFA intake in sustainable formulations. *TM* as a source of PUFAs opens promising avenues for studying its impact on lipid metabolism, inflammatory markers, and overall health in both clinical nutrition and sports performance contexts. Its potential use in omega‐3 fortification strategies, especially in populations with low fish consumption, makes it a valuable subject for future investigations focused on both nutritional efficacy and environmental sustainability (Orkusz 2021).

Regarding carbohydrates, these were not displayed on the powder label and this value was calculated by difference, but in the reviewed articles, they ranged from 10.8% (Sriprablom et al. 2022) to 11.45% (Son et al. 2021). The revised protein values ranged from 45.39% (Mazurek et al. 2024) to 54.6% (Zielińska et al. 2015) with our value also being an intermediate value (52.2%). Finally, the fiber content of our powder had lower values (3.7%) than those reviewed, 6.04% (Selaledi and Mabelebele 2021) to 11.92% (Mazurek et al. 2024). Although higher amounts of protein cannot be obtained with hydrolysate, it was very interesting to observe the protein/fat ratio in *TM* powder versus hydrolysate (1.6 vs. 5.1) in Table 3. A high protein‐to‐lipid ratio is particularly important in hydrolyzed insect powder, as it reflects an improved nutritional profile suitable for functional food applications. In this study, the hydrolysis process resulted in a marked reduction in lipid content, while protein levels were only moderately affected. This selective lipid reduction enhances the relative protein concentration of the final product, making it more attractive for use in high‐protein formulations and potentially improving the digestibility and bioavailability of insect‐derived proteins. Products with a high P/L (protein/lipid) ratio are especially desirable in dietary interventions targeting populations with increased protein needs—such as the elderly, individuals with sarcopenia, or those engaged in regular physical activity—while also aiming to limit excessive fat intake (Devries and Phillips 2015; Traylor et al. 2018).

No statistically significant differences in nutritional composition were observed between products DTM_1_H, DTM_1_HV, DTM_1_V as the variations in these products lie in the aromas used in their formulation, which only represented 0.1%–0.3% of the formula.

Among the brownie samples, BDTM_1_ had the lowest lipid content due to the absence of butter in its formulation, followed by BTM_2_, in which part of the *TM* powder was substituted with *TM* protein hydrolysate, which contains a lower lipid content. Additionally, BDTM_1_ did not include added sugar, resulting in a substantial reduction in carbohydrate content compared to BTM_1_ and BTM_2_. This reduction in lipids and carbohydrates impacts both the physicochemical and sensory properties of the product. Lower lipid content may lead to changes in texture and mouthfeel, while the absence of added sugar reduces sweetness and can affect color and flavor development due to diminished Maillard reactions. However, these compositional changes also offer potential health benefits by lowering the intake of fats and added sugars, aligning with current nutritional recommendations and potentially appealing to health‐conscious consumers.

The addition of *TM* protein hydrolysate to the brownie formula did not increase the protein and leucine content compared to the other formulations, due to the basic nutritional characteristics of this hydrolysate, which differs from that used in the study by Cho et al. (2020) with a protein content of 58% (Cho et al. 2020). Although the current *TM* protein hydrolysate extract may not exhibit a high degree of hydrolysis, its use remains highly relevant for future food and nutrition research. Protein hydrolysates, even at moderate hydrolysis levels, can provide improved digestibility, increased solubility, and enhanced functional properties such as emulsification and foaming capacity, which are valuable in complex food matrices (Hall et al. 2017). Furthermore, hydrolysis can lead to the generation of bioactive peptides with potential antioxidant, antihypertensive, or immunomodulatory properties—fields that are gaining attention in personalized and functional nutrition (Zielińska et al. 2018). Their potential to be tailored through controlled enzymatic treatment also opens the door to fine‐tuning their bioavailability and peptide profile for specific health outcomes (Bußler et al. 2016).

### 3.2 Physicochemical Properties

Table 4 shows the evaluation of physical properties of products with edible insects, namely the pasteurized dairy product with insect powder and the brownies with total substitution with insect powder, which will be described in the subsection shown below.

**TABLE 4:** Physicochemical characteristics of main ingredients used in D and brownie formulations.

Physical properties	D	*TM* (powder)	*TM* (hydrolysate)
Color attributes	L* 84.34 ± 0.33	L* 44.11 ± 0.45	L* 24.76 ± 0.01
	a* −2.85 ± 0.02	a* 8.04 ± 0.30	a* 0.75 ± 0.02
	b* 4.64 ± 0.44	b* 17.65 ± 0.35	b* −3.65 ± 0.05
Water activity	0.992 ± 0.0001	0.467 ± 0.0001	0.918 ± 0.003
pH	4.34 ± 0.01	6.25 ± 0.01	8.30 ± 0.01
Firmness (N)	1.16 ± 0.04	NA	NA
Cohesiveness (N)	−0.62 ± 0.01	NA	NA
Viscosity index (N)	−1.46 ± 0.04	NA	NA
Consistency index (N)	26.39 ± 0.00	NA	NA

*Note:* Results expressed in Mean ± SD.

Abbreviations: D, Pasteurized dairy product; N, Newtons; NA, not analyzed; *TM*: *Tenebrio molitor* .

#### 3.2.1 Physicochemical Properties of Pasteurized Dairy Product With Insect Powder ( *TM* )

The addition of *TM* powder to the D caused a decrease in % water and water activity and an increase in the firmness and index of cohesiveness. The incorporation of insect meal leads to an alkalization of the pH, which is similar to the results in Messina et al. (2019). In our study, the pH of the fermented milk was 4.34, whereas the average pH values for formulas DTM_1_V and DTM_1_H were recorded at 6.12, which was close to the pH of *TM* powder (6.25). Additionally, there was a decrease in the luminosity (L*) parameter and an increase in the values of color attributes a* and b*, with values in the formulated products much closer to *TM* powder than to D. The characteristics analyzed in Table 5 of the three D formulations with *TM* powder were similar. These results were expected if we consider that the only difference between them was the type of aroma (Table 5).

**TABLE 5:** Evaluation of physical properties of products with *TM*.

Physical properties	D with insect powder (DTM_1_)			Brownie with total substitution with edible insect (TM_1_; TM_2_)		
	DTM_1_H	DTM_1_V	DTM_1_HV	BDTM_1_	BTM_1_	BTM_2_
pH	6.14	6.11	6.11	NA	NA	NA
Water (%)	61.84 ± 0.05	57.13 ± 3.85	61.84 ± 1.63	53.19 ± 1.68^b^	35.25 ± 0.54^a^	34.59 ± 1.54^a^
Water activity	0.988	0.989	0.989	0.973 ± 0.001^c^	0.930 ± 0.011^b^	0.887 ± 0.001^a^
*Color attributes*						
L*	48.38 ± 0.09	48.23 ± 0.10	48.91 ± 0.62	41.24 ± 0.93^b^	25.20 ± 0.47^a^	28.37 ± 2.35^a^
a*	7.14 ± 0.22	7.04 ± 0.04	6.95 ± 0.11	11.82 ± 0.68^b^	5.20 ± 0.88^a^	6.18 ± 2.31^a^
b*	12.38 ± 0.29	12.82 ± 0.03	1.83 ± 0.21	22.58 ± 0.26^b^	4.73 ± 0.53^a^	6.73 ± 3.16^a^

Textural properties		Back extrusion (N)		TPA (N)		
Viscosity index	−75.2 ± 0.27	−78.34 ± 6.58	−71.54 ± 7.05	NA	NA	NA
Consistency index	472.99 ± 41.04	488.98 ± 35.66	493.44 ± 40.72	NA	NA	NA
Firmness	19.64 ± 0.50^b^	21.12 ± 0.03^a^	21.83 ± 0.31^a^	NA	NA	NA
Cohesiveness	−32.84 ± 0.06	−35.16 ± 0.45	−34.15 ± 1.49	0.30 ± 0.03^b^	0.20 ± 0.04^a^	0.37 ± 0.03^b^
Springiness	NA	NA	NA	0.39 ± 0.06^ab^	0.25 ± 0.02^a^	0.59 ± 0.14^b^
Chewiness	NA	NA	NA	0.38 ± 0.16^b^	0.42 ± 0.10^b^	2.69 ± 0.28^a^
Hardness	NA	NA	NA	3.17 ± 0.59^a^	8.18 ± 0.51^b^	12.74 ± 1.35^c^
Baking loss (%)	NA	NA	NA	13.6	10.0	5.7

*Note:* ^a–c^Different superscript in the same row and product typology indicate significant differences (*p* < 0.05). Results expressed in Mean ± SD.

Abbreviations: BDTM_1_, Brownie with pasteurized dairy product and *Tenebrio molitor* powder; BTM_1_, Brownie with *Tenebrio molitor* powder; BTM_2_, Brownie with *Tenebrio molitor* powder and *Tenebrio molitor* protein hydrolysate; DTM_1_H, Pasteurized dairy product with *Tenebrio molitor* powder and hazelnut aroma; DTM_1_HV, Pasteurized dairy product with *Tenebrio molitor* powder and hazelnut and vanilla aroma; DTM_1_V, Pasteurized dairy product with *Tenebrio molitor* powder and vanilla aroma.

#### 3.2.2 Physicochemical Properties of Brownies With Total Substitution With Insect Powder ( *TM* )

Brownies formulated with ingredients derived from edible insects exhibited differences in their physicochemical properties depending on the specific components used (Table 5). To further investigate these variations, pairwise comparisons were conducted to identify significant differences in instrumental physicochemical attributes between BTM_1_ and BTM_2_, as well as between BDTM_1_ and BTM_1_ (Table 6). *TM* powder altered the water content of the brownies depending on the amount used. When partially substituting traditional powder or eggs, the moisture content may slightly decrease, as insect powder does not retain water as effectively as wheat flour or cocoa powder (Halloran et al. 2016). *TM* protein hydrolysate exhibited higher water content and water activity compared to *TM* powder, due to the enzymatic hydrolysis involved in its production process. Additionally, its higher concentration of amino‐terminal groups contributes to a significantly elevated pH compared to *TM* powder (Kolakowski 2005). A comparison of the color parameters (L*, a*, b*) highlights the darker brown color of the hydrolysate in contrast to the lighter brown tone of *TM* powder.

**TABLE 6:** Significant differences of physicochemical properties between BTM_1_‐BTM_2_ and BDTM_1_‐BTM_1_.

Physicochemical properties	ANOVA, general test (*p*)	Product 1	Product 2	Tukey, pairwise comparison (*p*)
Water (%)	*p* = 0.001	BDTM_1_	BTM_1_	*p* = 0.002
Water activity	*p* = 0.002	BTM_1_	BTM_2_	*p* = 0.012
		BDTM_1_	BTM_1_	*p* = 0.012
L*	*p* < 0.001	BDTM_1_	BTM_1_	*p* < 0.001
a*	*p* = 0.003	BDTM_1_	BTM_1_	*p* = 0.003
b*	*p* < 0.001	BDTM_1_	BTM_1_	*p* < 0.001
Hardness	*p* < 0.001	BTM_1_	BTM_2_	*p* = 0.002
		BDTM_1_	BTM_1_	*p* = 0.001
Cohesiveness	*p* = 0.003	BTM_1_	BTM_2_	*p* = 0.002
		BDTM_1_	BTM_1_	*p* = 0.033
Springiness	*p* = 0.001	BTM_1_	BTM_2_	*p* = 0.008
Chewiness	*p* < 0.001	BTM_1_	BTM_2_	*p* < 0.001

*Note:* One‐way ANOVA and Tukey's post hoc test results, with a significance level of *α* = 0.05.

Abbreviations: BDTM_1_, Brownie with pasteurized dairy product and *Tenebrio molitor* powder; BTM_1_, Brownie with *Tenebrio molitor* powder; BTM_2_, Brownie with *Tenebrio molitor* powder and *Tenebrio molitor* protein hydrolysate.

BDTM_1_ had the highest water content, water activity, and % of baking loss. Its high baking loss was mostly related to its high initial water content and low lipid, carbohydrate, and protein content, with the last two components being relevant for water retention. BTM_1_ and BTM_2_ had similar water content, but BTM_2_ had a significantly lower water activity (*p* < 0.05). This could be related to the fact that the BTM_2_ formulation included *TM* protein hydrolysate, with more soluble proteins and a higher content of peptides and amino acids with lower molecular weight than *TM* powder, which contribute to water binding (Mishyna et al. 2021) and also explains the lower baking loss % of BTM_2_ compared to BTM_1_ (Çabuk 2021). Moreover, the lower baking loss in BTM_2_ could be related to the hydrolysate's ability to form a denser, more cohesive matrix that minimizes water evaporation during baking that would lead to a more efficient water retention within the protein network (Gani et al. 2015).

Regarding the measure of instrumental color, BDTM_1_, the formulation that included yogurt, egg, and egg white showed the highest value of L* since the darkest ingredients (*TM* powder and hydrolysate) were more diluted, or absent, in this formulation. The analysis of the color of BTM_2_ in our study yielded results similar to those published by Mshayisa et al. (2022). Moreover, the hydrolysate could have contributed to an intensified Maillard reaction, driven by its high concentration of free amino groups and low molecular weight peptides, resulting in a darker crust and crumb. Despite this, as will be detailed later, the darkest color of BTM_2_ was perceived as more appealing, likely due to associations with intense cocoa flavor and traditional brownie appearance, aligning with studies that indicate darker baked goods are often preferred for their perceived richness (Dossey et al. 2016).

Textural properties of brownie formulations were analyzed. BDTM_1_ was the formula with the lowest values of hardness and chewiness, followed by BTM_1_ while BTM_2_ had the highest values of these parameters. According to Ho et al. (2022), who studied brownie formulations that included cricket powder, brownie hardness and chewiness are negatively correlated with water content. Their observation is consistent with the results of our study since BDTM_1_ water content was around 53%, while the water content of BTM_1_ samples was around 35%. Nonetheless, although BTM_1_ and BTM_2_ had the same water content, BTM_2_ showed significantly higher values of chewiness and hardness. This difference could be related to the lower fat content of BTM_2_ compared to BTM_1_ as fat contributes to cake tenderness (Cauvain and Young 2008). Since instrumental chewiness is the result of multiplying the values of hardness, cohesiveness, and springiness (Meullenet et al. 1998) the high values of BTM_2_ chewiness are mostly related to the high hardness of this sample.

BTM_2_ also showed high values for springiness (elasticity) and cohesiveness. Cohesiveness measures the ability to maintain structural integrity when subjected to mechanical forces (such as chewing) (Bourne 1982). Values observed in this study are similar to those reported in muffins formulated with 100% mealworm powder (Zielińska et al. 2018). These values are lower than formulations with wheat flour since mealworm powder lacks wheat gluten structuring properties. BDTM_1_ and BTM_2_ had greater cohesiveness compared to BTM_1_. On one hand, these samples had a lower percentage of insect powder than BTM_2_. On the other hand, BDTM_1_ included D proteins and an increased level of egg white proteins compared to BTM_1_, while BTM_2_ included *TM* protein hydrolysate. The functionality of these proteins could have improved the cohesiveness of BDTM_1_ and BTM_2_ compared to BTM_1_ with egg yolk and protein hydrolysate contributing to better aeration and structural stability. The higher protein and fiber content of insect powder promotes water competition and reduces hydration, creating a denser, less extensible matrix. These structural changes explain the increase in perceived hardness and mechanical resistance during mastication (Luna et al. 2021; Purschke et al. 2018). Additionally, the hydrolysate contribution to strong protein–protein interactions during baking results in a firm, elastic, and highly structured matrix, significantly increasing hardness and chewiness. This aligns with previous findings where hydrolyzed insect proteins induced network densification and increased mechanical resistance under compression (Purschke et al. 2018). Interestingly, although cohesiveness remained moderate, this might suggest that consumers could tolerate reduced softness as long as the matrix remains stable and intact during mastication. The hydrolysate may enhance protein foaming and stabilization properties, resulting in better crumb aeration (Purschke et al. 2018). However, this benefit did not fully compensate for the reduced acceptability driven by bitterness and lack of sweetness, as will be discussed next. These findings confirm that variations in moisture, fat, and protein type play a crucial role in modulating the mechanical characteristics of the brownies, as observed by Ho et al. (2022), who demonstrated that alterations in formulation composition can lead to measurable differences in the texture and moisture content of brownies formulated with insect powder. Similarly, Kowalski et al. (2022) who studied the effect of different concentrations of insect powders, observed changes in the textural parameters of wheat bread.

The brownie made with pasteurized dairy product contained 7% less *TM* powder than BTM_1_, leading to significant physicochemical differences.

### 3.3 Sensory Evaluation

**FIGURE 3:**
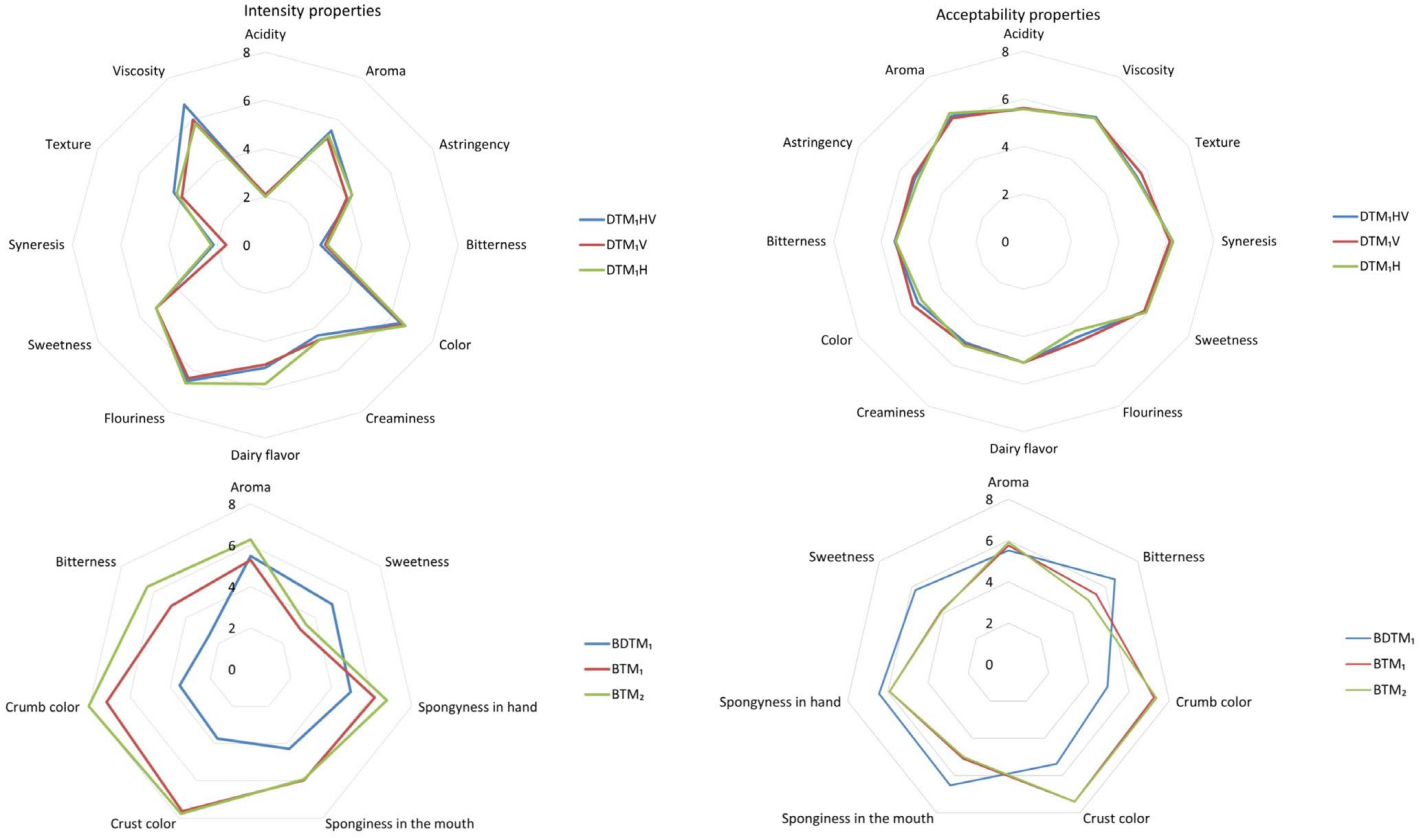
Spider plots of the sensory properties of CATA 1 and CATA 2. CATA 1: DTM_1_H, Pasteurized dairy product with *Tenebrio molitor* powder and hazelnut aroma; DTM_1_HV, Pasteurized dairy product with *Tenebrio molitor* powder and hazelnut and vanilla aroma; DTM_1_V, Pasteurized dairy product with *Tenebrio molitor* powder and vanilla aroma; CATA 2: BDTM_1_, Brownie with pasteurized dairy product and *Tenebrio molitor* powder; BTM_1_, Brownie with *Tenebrio molitor* powder. BTM_2_, Brownie with *Tenebrio molitor* powder and *Tenebrio molitor* protein hydrolysate.

The bivariate correlation study revealed a positive, albeit weak, correlation (*r* = 0.232, *p* < 0.05) between the participants' levels of neophobia and their evaluation of the product's overall product appearance. This suggests that there is some tendency for individuals with higher neophobia scores to rate the global aspect of the product differently from those with lower neophobia scores. However, the correlation coefficient indicates a relatively weak association, implying that other factors may also play a significant role in shaping the participants' evaluation of the global aspect of the product. The results of the ANOVA tests comparing three products (DTM_1_H, DTM_1_HV, DTM_1_V) in terms of intensity and acceptability attributes indicate that there were no significant differences among them. All the *p*‐values of the variables studied were higher than the significance level (*α* = 0.05), suggesting that there was no strong evidence to support significant variations in these attributes among the products. These results can be visually observed in the spider plots in Figure 3, where the intensity and acceptability attributes for the three products (DTM_1_H, DTM_1_HV, DTM_1_V) are represented on a hedonic scale ranging 1–9 (Wichchukit and O'Mahony 2015).

When queried about the best‐rated product, responses yielded scores of DTM_1_HV (52.4%), DTM_1_V (23.8%), and DTM_1_H (23.8%), with no significant differences. However, when assessing the least preferred product, significant differences emerged, with values of DTM_1_V (61.9%), DTM_1_HV (19%), and DTM_1_H (19%) (Table S1).

In the brownie with total substitution with insect powder (BTM_1_), the neophobia scale used shows good reliability (Cronbach's alpha = 0.72). The overall means of the food habits questions responses ranged 1.2–3.3. There is no correlation between neophobia scores and the final product evaluation (coefficient = −0.02).

Responses to the inquiry about the best‐rated product were as follows: BDTM_1_ (56%), BTM_1_ (20%), and BTM_2_ (24%) (Table S2).

In the sensory evaluation analysis of the formulations DTM_1_H, DTM_1_HV, DTM_1_V, no significant differences were found (Table 7 and Figure 3).

**TABLE 7:** Sensory attributes of products with *TM*.

Sensory attributes	D with insect powder (DTM_1_)			Brownie with total substitution with edible insect (TM_1_; TM_2_)		
	DTM_1_H	DTM_1_V	DTM_1_HV	BDTM_1_	BTM_1_	BTM_2_
Syneresis Intensity Acceptability	2.24 ± 2.28 6.29 ± 1.95	1.62 ± 1.83 6.14 ± 1.98	2.14 ± 2.03 6.29 ± 1.82	NA	NA	NA
Viscosity Intensity Acceptability	5.81 ± 2.69 6.00 ± 1.58	6.00 ± 2.76 6.00 ± 1.41	6.71 ± 2.19 6.05 ± 1.50	NA	NA	NA
Color Intensity Acceptability	6.71 ± 1.38 4.95 ± 1.91	6.62 ± 1.40 5.38 ± 1.80	6.48 ± 1.63 5.14 ± 1.80	NA	NA	NA
Aroma Intensity	5.24 ± 2.05	5.14 ± 1.88	5.48 ± 1.86	5.48 ± 1.73	5.28 ± 2.26	6.28 ± 1.93
Acceptability	6.24 ± 1.64	6.00 ± 1.26	6.10 ± 1.14	5.52 ± 2.45	5.76 ± 2.09	5.92 ± 1.93
Sweetness Intensity	5.24 ± 1.45	5.24 ± 1.84	5.24 ± 1.87	5.04 ± 1.67^b^	3.08 ± 1.53^a^	3.44 ± 2.14^a^
Acceptability	5.95 ± 0.97	5.86 ± 1.20	5.86 ± 1.06	5.76 ± 2.33	4.16 ± 2.32	4.12 ± 2.33
Bitterness Intensity	2.57 ± 1.83	2.48 ± 1.60	2.29 ± 1.55	2.60 ± 1.71^a^	4.92 ± 1.80^b^	6.40 ± 1.47^c^
Acceptability	5.38 ± 1.32	5.38 ± 1.53	5.43 ± 1.29	6.60 ± 2.55	5.44 ± 2.58	4.96 ± 2.46
Astringency Intensity Acceptability	4.14 ± 2.24 5.14 ± 1.74	3.90 ± 2.26 5.38 ± 1.60	4.14 ± 2.50 5.29 ± 1.74	NA	NA	NA
Texture Intensity Acceptability	4.24 ± 2.36 5.43 ± 1.08	4.00 ± 2.10 5.71 ± 1.42	4.38 ± 2.44 5.48 ± 1.03	NA	NA	NA
Flouriness Intensity Acceptability	6.62 ± 2.01 4.33 ± 1.91	6.38 ± 2.01 4.81 ± 1.83	6.52 ± 1.89 4.62 ± 1.66	NA	NA	NA
Creaminess Intensity Acceptability	4.52 ± 2.27 5.05 ± 1.53	4.52 ± 2.11 5.00 ± 1.41	4.33 ± 2.11 4.90 ± 1.37	NA	NA	NA
Dairy flavor Intensity Acceptability	5.76 ± 1.48 5.10 ± 0.3	4.95 ± 0.8 5.10 ± 0.3	5.10 ± 1.55 5.10 ± 0.3	NA	NA	NA
Crumb color Intensity Acceptability	NA	NA	NA	3.52 ± 1.05^a^	7.16 ± 1.34^b^	8.04 ± 1.02^c^
				4.92 ± 2.31^a^	7.24 ± 1.33^b^	7.36 ± 1.52^b^
Crust color Intensity Acceptability	NA	NA	NA	3.72 ± 1.51^a^	7.64 ± 1.08^b^	7.76 ± 1.30^b^
				5.36 ± 2.22^a^	7.40 ± 1.38^b^	7.40 ± 1.73^b^
Sponginess in mouth Intensity Acceptability	NA	NA	NA	4.28 ± 2.07^a^	5.96 ± 2.07^b^	5.92 ± 1.93^b^
				6.52 ± 2.40	5.08 ± 2.33	5.00 ± 2.27

*Note:* ^a–c^Different superscript in Mean ± SD in the same row and product typology with different superscript indicates that there are significant differences (*p* < 0.05). Results expressed in Mean ± SD.

Abbreviations: BDTM_1_, Brownie with pasteurized dairy product and *Tenebrio molitor* powder; BTM_1_, Brownie with *Tenebrio molitor* powder; BTM_2_, Brownie with *Tenebrio molitor* powder and *Tenebrio molitor* protein hydrolysate; DTM_1_H, Pasteurized dairy product with *Tenebrio molitor* powder and hazelnut aroma; DTM_1_HV, Pasteurized dairy product with *Tenebrio molitor* powder and hazelnut and vanilla aroma; DTM_1_V, Pasteurized dairy product with *Tenebrio molitor* powder and vanilla aroma.

In the brownies, the color intensity of the BDTM_1_ formula corresponded to its highest L* values. Probably, the panelists expected a richer color in the brownie formulas; hence, the acceptability score of BDTM_1_ with respect to BTM_2_ and BTM_1_ is considered fairer. This aspect needs consideration for the future of such formulations, as the stronger color has better acceptability in BTM_2_ and BTM_1_ products than in BDTM_1_, suggesting that the brownie formula, already associated with a darker color (Hensley 2021), may be well suited for working with this type of powder (Table 7).

The aroma (intensity of cocoa) of the three formulations achieved an average score of 5.5 in terms of intensity and was positively accepted, with an average score of 5.7, without significant differences between them. Concerning sweetness, although the BDTM_1_ formula lacked added sugar, it was perceived to have a higher intensity of sweetness compared to the other two formulas, BTM_2_ and BTM_1_, even if they had sugar at respective amounts of 11.6 g. This suggests that the D product (with residual lactose and saccharose) (Nsofor and Frank 2012) and essence, containing vanilla, orange peel, and orange blossom aroma, may have helped the BDTM_1_ formula improve in sweetness without the need to incorporate sugar, representing a potential area for future research. In the case of BDTM_1_, the incorporation of D resulted in a more aerated formulation.

As expected, bitterness was more intense in the BTM_2_ formula due to the inherently bitter nature of insect hydrolysate, which may contain bitter peptides and amino acids. Simultaneously, the acceptability for this product and this particular parameter was found to be the lowest. The pronounced bitterness observed in the BTM_2_ is likely due to the accumulation of hydrophobic and low‐molecular‐weight peptides produced during enzymatic hydrolysis (Schlegel et al. 2019). These peptides are known to activate bitter taste receptors and have been reported as one of the main sensory drawbacks of protein hydrolysates. This bitter taste significantly reduced sweetness perception and overall hedonic acceptability, as bitterness can suppress sweetness through central taste interactions (Fu et al. 2019).

Intensity of sponginess in the hand obtained the highest values for the BTM_1_ and BTM_2_ formulas, compared to BDTM_1_, but acceptability was the lowest in these two. This could be due to the fact that more compact structures are expected with the brownie, and therefore sponginess in the brownie is not a parameter that is sought as more acceptable (Candela 2021). This difference in sponginess between BTM_2_, BTM_1_, and BDTM_1_ could be related to the lower fat content of BDTM_1_. These results suggest a negative correlation between insect powder concentration and perceived sponginess.

Sensory attributes, the crumb and crust color intensities, were lower in BDTM_1_ compared to BTM_1_, which correlated with reduced acceptability scores. However, the higher sweetness intensity in BDTM_1_ contributed to better overall acceptability. Bitterness intensity was more pronounced in BTM_1_ than in BDTM_1_, while the sponginess intensity was lower in BDTM_1_ compared to BTM_1_, highlighting notable differences in the sensory profile between the two formulations.

An evaluation was performed to detect significant pairwise differences in sensory properties between BTM_1_‐BTM_2_ and BDTM_1_‐BTM_1_ (Table 8).

**TABLE 8:** Differences of sensory attributes between BTM_1_‐BTM_2_ and BDTM_1_‐BTM_1_.

Sensory attributes	ANOVA, general test (*p*)	Product 1	Product 2	Tukey, pairwise comparison (*p*)
Intensity of crumb color	*p* < 0.001	BTM_1_	BTM_2_	*p* = 0.022
		BDTM_1_	BTM_1_	*p* < 0.001
Acceptance of crumb color	*p* < 0.001	BDTM_1_	BTM_1_	*p* < 0.001
Intensity of crust color	*p* < 0.001	BDTM_1_	BTM_1_	*p* < 0.001
Acceptance of crust color	*p* < 0.001	BDTM_1_	BTM_1_	*p* < 0.001
Intensity of sweetness	*p* < 0.001	BDTM_1_	BTM_1_	*p* < 0.001
Acceptance of sweetness	*p* = 0.022	BDTM_1_	BTM_1_	*p* = 0.046
Intensity of bitterness	*p* < 0.001	BTM_1_	BTM_2_	*p* = 0.007
		BDTM_1_	BTM_1_	*p* < 0.001
Intensity of sponginess in mouth	*p* = 0.006	BDTM_1_	BTM_1_	*p* = 0.012

*Note:* One‐way ANOVA and Tukey's post hoc test results, with a significance level of α = 0.05.

Abbreviations: BDTM_1_, Brownie with pasteurized dairy product and *Tenebrio molitor* powder; BTM_1_, Brownie with *Tenebrio molitor* powder; BTM_2_, Brownie with *Tenebrio molitor* powder and *Tenebrio molitor* protein hydrolysate.

**FIGURE 4:**
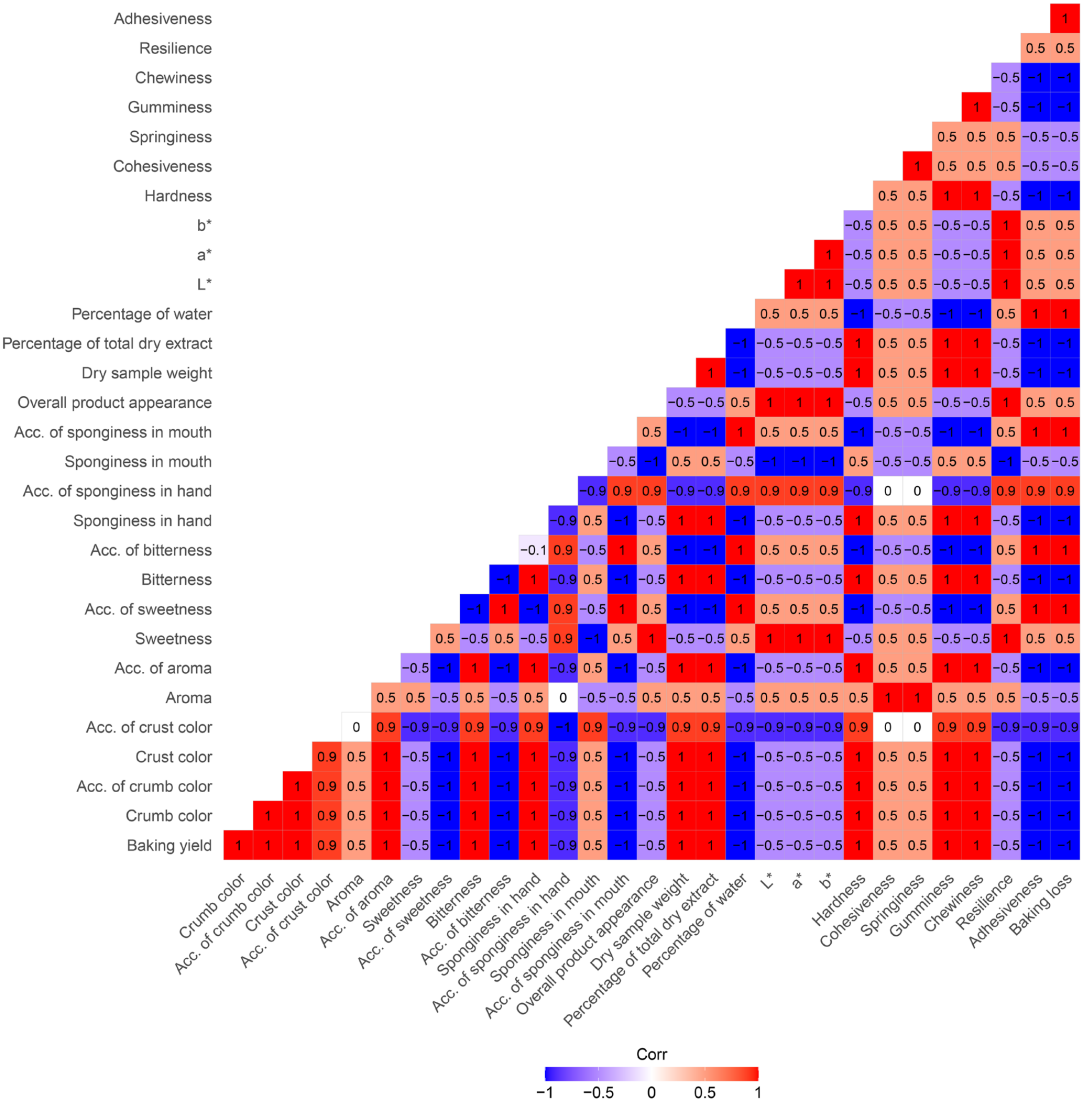
Correlations of physicochemical and sensory properties between the products with *TM* ( *Tenebrio molitor* ).

High positive correlations were observed between the acceptability of crumb color and crust color across all three brownie formulas, with values ranging from *r* = 0.72 for BTM_1_, *r* = 0.75 for BDTM_1_, and *r* = 0.80 for BTM_2_. These findings suggest that consumers evaluate these two visual attributes similarly, emphasizing the importance of color consistency in both the crumb and crust for enhancing overall product evaluation. Additionally, the correlation between crust color acceptability and overall crust color was moderate, with values of *r* = 0.61 for BDTM_1_, *r* = 0.57 for BTM_1_ and *r* = 0.57 for BTM_2_. This indicates that while the visual appeal of crust color significantly influences product acceptance, its impact may be somewhat less pronounced compared to the combined evaluation of crumb and crust colors. Together, these results highlight the critical role of visual attributes in shaping consumer perceptions and preferences for brownie formulations (Figure 4).

Furthermore, the correlation between global product aspect and sweetness acceptability ranged from *r* = 0.53 for BDTM_1_, *r* = 0.59 for BTM_1_, to *r* = 0.67 for BTM_2_. This indicates that a satisfactory level of sweetness contributes significantly to the overall positive evaluation of the product.

The two most highly correlated attributes observed across the three brownie formulas were the acceptability of sponginess in hand and the acceptability of sponginess in mouth. The highest correlation was seen in the BDTM_1_ formula, with an impressive correlation coefficient of *r* = 0.91. For the BTM_2_ formula, the correlation was *r* = 0.65, while for the BTM_1_ formula, it was *r* = 0.76. This indicates that consumers who perceive the brownie as spongy in hand also tend to find it equally spongy in the mouth, regardless of the formula.

These results show that for the BDTM_1_ formula, consumers who rate the product as spongy in the mouth or in hand are more likely to evaluate the overall product positively. However, this pattern does not hold for the other two formulas (BTM_1_ and BTM_2_), where the correlation between sponginess and global product evaluation is weaker.

**FIGURE 5:**
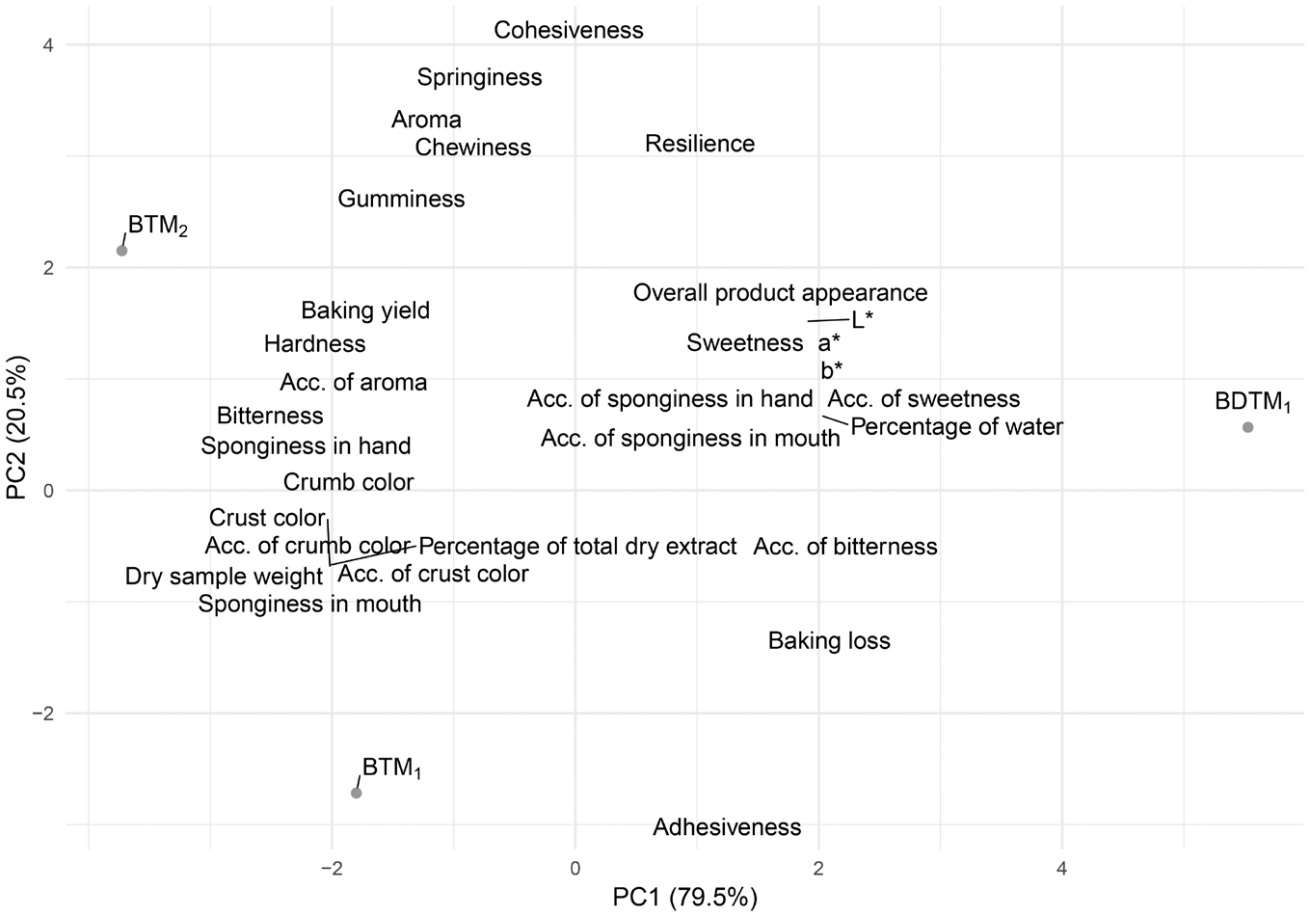
Principal component analysis between BDTM_1_, BTM_1_ and BTM_2_. BTM_1_, Brownie with *Tenebrio molitor* powder; BTM_2_, Brownie with *Tenebrio molitor* powder and *Tenebrio molitor* protein hydrolysate.

PC1 is primarily explained by attributes such as overall product appearance, color properties (a*, b*, L*), acceptability of sponginess in hand, acceptability of sponginess in mouth, acceptability of bitterness, and baking loss, among others. The product BDTM_1_ exhibited higher values for these attributes. In contrast, the other two products (BTM_2_ and BTM_1_) showed higher values for the variables positioned on the left side of the graph, such as baking yield, hardness, sponginess in hand, crumb color, and others (Figure 5). PCA revealed strong associations between lipid content and specific sensory attributes such as texture and mouthfeel, offering insights into how formulations based on *TM* powder and *TM* hydrolysate influenced product perception. The first two principal components together explained 100% variance (PC1 = 79.5%, PC2 = 20.5%).

The greater the separation between products on the plot, the more distinct they are. It is evident that BTM_2_ and BTM_1_ are more similar to each other compared to their relationship with BDTM_1_ (as previously observed in spider plots, Figure 3).

In summary, the sensory evaluations revealed that the incorporation of hydrolyzed or powdered *TM* influences key attributes such as bitterness, sweetness perception, sponginess, and color, which in turn affect overall product acceptability. The use of insect powder at levels above 33% led to less favorable physicochemical and sensory properties—such as increased bitterness and hardness—compared to formulations containing *TM* protein hydrolysate, which showed improved cohesiveness and elasticity. While neophobia appeared to have only a weak influence on product evaluation, visual attributes (especially crust and crumb color) and perceived sweetness showed strong positive correlations with hedonic responses. These findings emphasize the importance of balancing functional and sensory elements in insect‐enriched formulations and suggest that thoughtful matrix selection, such as brownies, may enhance consumer acceptance of such innovative protein sources.

While this study provides a comprehensive assessment of the physicochemical, nutritional, and sensory properties of insect‐enriched formulations, several aspects offer promising avenues for future research. For instance, expanding the number and diversity of participants in sensory evaluations could enhance the robustness and applicability of the findings across broader consumer groups. The inclusion of a control formulation based exclusively on conventional wheat flour may also provide valuable benchmarks for interpreting the specific contributions of insect‐derived ingredients. Additionally, exploring different degrees of protein hydrolysis could help optimize peptide availability and functional performance within the matrix. Future studies might also consider evaluating protein digestibility and bioavailability to deepen understanding of the nutritional potential of insect‐enriched products. These perspectives may help guide the development and positioning of insect‐based ingredients in food innovation.

## 4 Conclusions

Insect‐derived proteins, particularly from *TM*, offer substantial nutritional benefits in terms of protein quality, energy density, and essential amino acid content, while aligning with long‐term sustainability goals. This study demonstrates that while insect powder can be successfully incorporated into food matrices such as brownies, inclusion levels exceeding approximately 30% may negatively affect physicochemical properties—leading to increased hardness, reduced cohesiveness, diminished sweetness perception, and compromised acceptability. The integration of *TM* protein hydrolysate significantly improved cohesiveness, elasticity, and chewiness, offering a functional solution for reaching target protein and leucine levels without deteriorating structural or sensory quality. These enhancements are particularly relevant for populations with increased protein needs, such as older adults or individuals with chewing difficulties. In terms of future formulations, the use of insect hydrolysates seems the best option given the results obtained, as their incorporation allows an increase in protein content without excessively increasing fat content, thus offering a more balanced macronutrient profile. Furthermore, sensory trials revealed that hazelnut and vanilla flavorings improved overall acceptability, suggesting that thoughtful gastronomic formulation is essential to overcome neophobic barriers. Despite the challenges in texture and bitterness at high insect protein levels, consumer expectations regarding darker color, compact structure, and moderate height were consistent with product characteristics. Given the limited existing literature on hydrolyzed insect proteins in bakery products, future research should explore optimized formulations and sensory strategies to improve familiarity and consumer acceptance of insect‐enriched functional foods in Western diets.

## Author Contributions

**Marta Ros‐Baró:** conceptualization (equal), data curation (equal), formal analysis (equal), investigation (equal), methodology (equal), resources (equal), software (equal), validation (equal), writing – original draft (equal), writing – review and editing (equal). **Marta Capellas Puig:** conceptualization (equal), data curation (equal), formal analysis (equal), methodology (equal), writing – original draft (equal), writing – review and editing (equal). **Gemma Chiva‐Blanch:** data curation (equal), validation (equal), writing – original draft (equal), writing – review and editing (equal). **Diana A. Díaz‐Rizzolo:** validation (equal), writing – original draft (equal), writing – review and editing (equal). **Alicia Aguilar Martínez:** validation (equal), writing – original draft (equal), writing – review and editing (equal). **Montserrat Jorba Rafart:** validation (supporting), writing – original draft (supporting), writing – review and editing (supporting). **Irina Chiriac:** validation (equal), writing – original draft (equal), writing – review and editing (equal). **Mar Blanco Rogel:** validation (supporting), writing – original draft (supporting), writing – review and editing (supporting). **Montserrat Pujolà Cunill:** conceptualization (equal), data curation (equal), methodology (equal), resources (equal), software (equal), validation (equal), writing – original draft (equal), writing – review and editing (equal). **Patricia Casas‐Agustench:** conceptualization (equal), data curation (equal), formal analysis (equal), investigation (equal), methodology (equal), resources (equal), software (equal), supervision (equal), validation (equal), writing – original draft (equal), writing – review and editing (equal). **Anna Bach‐Faig:** conceptualization (equal), data curation (equal), formal analysis (equal), investigation (equal), methodology (equal), resources (equal), software (equal), supervision (equal), validation (equal), writing – original draft (equal), writing – review and editing (equal).

## Ethics Statement

Ethical approval was obtained from the ethics committee of UOC University (Universitat Oberta de Catalunya). Ethics Committee (Exp.: ce23‐TE33).

## Consent

Written informed consent was obtained from all study participants.

## Conflicts of Interest

The authors declare no conflicts of interest.

## Supporting information


**Table S1:** Statistics of final product valorization CATA 1.
**Table S2:** Statistics of final product valorization CATA 2.

## Data Availability

Data will be made available upon reasonable request.
