# Supplementary material for: Enrichment of Pasteurized Dairy Product and Brownie With Edible Insect ( Tenebrio molitor ) to Analyze Acceptance Using Check‐All‐That‐Apply Methodology
Source: Food Sci Nutr. 2025 Oct 30;13(11):e70925. doi: 10.1002/fsn3.70925 (PMC12576023; doi:10.1002/fsn3.70925)
Supplement: Supplementary file 1 — Table S1: Statistics of final product valorization CATA 1. Table S2: Statistics of final product valorization CATA 2. [file FSN3-13-e70925-s001.docx]

**SUPPLEMENTARY TABLE 1:** Statistics of final product valorization CATA 1.

| **Product valorization** | **DTM_1_HV** | **DTM_1_V** | **DTM_1_H** | **p-value** |
| --- | --- | --- | --- | --- |
| **Best** | 11 (52.4%) | 5 (23.8%) | 5 (23.8%) | 0.18 |
| **Medium** | 12 (57.1%) | 3 (14.3%) | 6 (28.6%) | **0.04** |
| **Worst** | 4 (19.0%) | 13 (61.9%) | 4 (19.0%) | **0.02** |

Note: One-way ANOVA post hoc test results, with a significance level of α=0.05. DTM_1_H: Pasteurized dairy product with *Tenebrio molitor* powder and hazelnut aroma; DTM_1_HV: Pasteurized dairy product with *Tenebrio molitor* powder and hazelnut and vanilla aroma; DTM_1_V: Pasteurized dairy product with *Tenebrio molitor* powder and vanilla aroma.

**SUPPLEMENTARY TABLE 2:** Statistics of final product valorization CATA 2.

| **Product valorization** | **BDTM_1_** | **BTM_1_** | **BTM_2_** | **p-value** |
| --- | --- | --- | --- | --- |
| **Best** | 14 (56%) | 5 (20%) | 6 (24%) | **0.05** |
| **Medium** | 7 (28%) | 12 (48%) | 6 (24%) | 0.29 |
| **Worst** | 4 (16%) | 8 (32%) | 13 (52%) | 0.09 |

Note: One-way ANOVA post hoc test results, with a significance level of α=0.05. Abbreviations: BDTM_1_: Brownie with pasteurized dairy product and *Tenebrio molitor* powder; BTM_1_: Brownie with *Tenebrio molitor* powder; BTM_2_: Brownie with *Tenebrio molitor* powder and *Tenebrio molitor* protein hydrolysate.
